# Supplementary material for: Complexome profiling of the Chlamydomonas psb28 mutant reveals TEF5 as an early PSII assembly factor
Source: Plant Cell. 2025 Mar 17;37(6):koaf055. doi: 10.1093/plcell/koaf055 (PMC12164590; doi:10.1093/plcell/koaf055)
Supplement: koaf055_Supplementary_Data [file koaf055_supplementary_data.zip › psb28_interactive.zip!/plots/intensity/qualityPlots/peptides/OGD3_Cre03.g185600.t1.1.html]

### Cre03.g185600.t1.1

Trivial name: OGD3  
SeqCoverage [median]: 36.60  
Mass: 22.00 kDa  
  
 

| Sequence | MissedCleavage | Mass [Da] | wtIntensity | muIntensity | Score |
| --- | --- | --- | --- | --- | --- |
| APAAGFLHK | 0 | 910.50 | 741.74 | 0.00 | 62.71 |
| EGTIAAVLK | 0 | 900.53 | 376.00 | 3332.39 | 73.26 |
| ELELINLGGAQ | 0 | 1155.61 | 1814.40 | 0.00 | 234.71 |
| ISSLSDGEYEK | 0 | 1226.57 | 2539.71 | 13113.19 | 154.05 |
| LTYVTRPDGR | 1 | 1176.63 | 8973.05 | 21887.03 | 59.43 |
| PAVAPHPMSAMK | 0 | 1235.62 | 372.21 | 0.00 | 49.19 |
| PDVVVPPMGESIKEGTIAAVLK | 1 | 2249.23 | 752.75 | 5313.72 | 47.75 |
| QVGAAVR | 0 | 699.40 | 0.00 | 425.26 | 69.43 |
|  |  |  | 15569.9 | 44071.6 |  |
